# A new *Plasmodium vivax* reference genome for South American isolates

**DOI:** 10.1186/s12864-023-09707-5

**Published:** 2023-10-11

**Authors:** Katlijn De Meulenaere, Bart Cuypers, Dionicia Gamboa, Kris Laukens, Anna Rosanas-Urgell

**Affiliations:** 1 Department of Biomedical Sciences, Institute of Tropical Medicine Antwerp, Antwerp, Belgium; 2 Department of Computer Science, University of Antwerp, Antwerp, Belgium; 3 Instituto de Medicina Tropical Alexander von Humboldt, Universidad Peruana Cayetano Heredia, Lima, Peru; 4 Departamento de Ciencias Celulares y Moleculares, Facultad de Ciencias y Filosofía, Universidad Peruana Cayetano Heredia, Lima, Peru

**Keywords:** *Plasmodium vivax*, Genome assembly, PacBio sequencing, Reference genome

## Abstract

**Background:**

*Plasmodium vivax* is the second most important cause of human malaria worldwide, and accounts for the majority of malaria cases in South America. A high-quality reference genome exists for Papua Indonesia (PvP01) and Thailand (PvW1), but is lacking for South America. A reference genome specifically for South America would be beneficial though, as *P. vivax* is a genetically diverse parasite with geographical clustering.

**Results:**

This study presents a new high-quality assembly of a South American *P. vivax* isolate, referred to as PvPAM (*P. vivax* Peruvian AMazon). The genome was obtained from a low input patient sample from the Peruvian Amazon and sequenced using PacBio technology, resulting in a highly complete assembly with 6497 functional genes. Telomeric ends were present in 17 out of 28 chromosomal ends, and additional (sub)telomeric regions are present in 12 unassigned contigs. A comparison of multigene families between PvPAM and the PvP01 genome revealed remarkable variation in *vir* genes, and the presence of merozoite surface proteins (MSP) 3.6 and 3.7. Three *dhfr* and *dhps* drug resistance associated mutations are present in PvPAM, similar to those found in other Peruvian isolates. Mapping of publicly available South American whole genome sequencing (WGS) data to PvPAM resulted in significantly fewer variants and truncated reads compared to the use of PvP01 or PvW1 as reference genomes. To minimize the number of core genome variants in non-South American samples, PvW1 is most suited for Southeast Asian isolates, both PvPAM and PvW1 are suited for South Asian isolates, and PvPAM is recommended for African isolates. Interestingly, non-South American samples still contained the least subtelomeric variants when mapped to PvPAM, indicating high quality of the PvPAM subtelomeric regions.

**Conclusions:**

Our findings show that the PvPAM reference genome more accurately represents South American *P. vivax* isolates in comparison to PvP01 and PvW1. In addition, PvPAM has a high level of completeness, and contains a similar number of annotated genes as PvP01 or PvW1. The PvPAM genome therefore will be a valuable resource to improve future genomic analyses on *P. vivax* isolates from the South American continent.

**Supplementary Information:**

The online version contains supplementary material available at 10.1186/s12864-023-09707-5.

## Background

*Plasmodium vivax* is the second most common cause of malaria in humans after *P. falciparum*. *P. vivax* is widespread outside of Africa, accounting there for one-third of the malaria infections [1]. Due to the absence of a continuous culturing system [2] and the former assumption that *P. vivax* infections are benign [3], *P. vivax* research has been lagging behind that of *P. falciparum*. Only in 2008, the first *P. vivax* reference genome, Salvador I, was assembled [4], while the *P. falciparum* 3D7 reference genome was already published in 2002 [5].

Hitherto, several *P. vivax* genomes have been assembled for *P. vivax*. The Salvador I (PvSalI) genome was assembled from paired-read Sanger sequencing data, coming from an El Salvador isolate that was passaged in *Saimiri* monkeys [4, 6]. However, it contains > 2000 unassigned contigs that were not assigned to a chromosome, making it less suited for alignment of whole genome sequencing (WGS) data. Similarly, Illumina-based assemblies from four monkey-passaged isolates from Brazil, India, Mauritania and North Korea [7] and 1 Cambodian field isolate [8] were highly fragmented, with > 1500 scaffolds. More recently, Auburn et al. (2016) released a high-quality reference genome from an Illumina-sequenced Papua Indonesian isolate (PvP01), along with two draft assemblies from Thailand (PvT01) and China (PvC01). Finally, a Thai isolate used in controlled human infections was long-read sequenced with PacBio, resulting in the high-quality PvW1 reference genome [9].

South America is not yet represented by the existing high-quality reference genomes (PvP01, PvW1), although the majority of the malaria cases in this continent are *P. vivax* [1]. Since *P. vivax* is genetically more diverse than *P. falciparum* [7, 10, 11], there is a strong geographical differentiation between isolates from different continents [11–13]. The PvP01 and PvW1 assemblies originate from Papua Indonesia and Thailand, and isolates from these countries are genetically distant from the South American ones [12]. Therefore, a new *P. vivax* reference genome from South American origin could improve variant calling of South American isolates and provide more accurate information on indels (insertions and deletions) and large structural variants. Especially these large structural variants cannot be detected readily by aligning short-read sequences to a reference genome, which is why continent-specific reference genomes are expected to increase mapping quality and reveal new or missing genes. This will have a greater impact in the subtelomeric regions, which are highly dynamic with frequent duplication and recombination events [14], and contain important multigene families involved in immune evasion and host-parasite interactions [4]. In addition, accurate reflection of the sample’s structural variants in the reference genome can help the assessment of RNA isoforms, which are known to be abundant in *P. vivax* [15–18].

Since *de novo* assembly of a reference genome makes use of the overlaps between sequenced fragments (reads), longer reads improve confidence and quality of the assembly. In addition, long reads can better resolve repetitive or low-complexity regions often found in the subtelomeres. Short-read technologies like Illumina typically have read lengths of 50–300 bases, while Pacific Biosciences SMRT technology (PacBio) and Oxford Nanopore technology (ONT), the leading long-read sequencing technologies to date, readily exceed 10,000 bases. Therefore, PacBio and Nanopore are more suited for reference genome sequencing. At the moment of writing, PacBio sequencing has an accuracy of 99.8% [19], similar to what can be achieved with Illumina sequencing, and higher than the 99% that can be reached with the Nanopore R10.4 chemistry [20]. Due to PacBio’s use of circular consensus sequencing (a circular read is sequenced multiple times), random sequencing errors are largely removed [19]. Although a bias for indels at homopolymer regions remains [19, 21], it is the most robust long-read sequencing method to date, which was also used to construct the Thai PvW1 reference [9]. The PvW1 sample was obtained from controlled human malaria infections, which allows extraction of a high amount of input DNA. However, low input protocols are becoming more common, and PacBio recently released their Ultra-Low DNA Input protocol (5–20 ng DNA) [22]. This provides opportunities to long-read sequence *P. vivax* DNA from low blood volume and/or low parasitaemia patient samples, which is common in natural human infections.

In this study, a leukocyte-depleted blood sample from a *P. vivax* patient from the Peruvian Amazon was sequenced with PacBio using the Ultra-Low DNA Input protocol, allowing the assembly of a new and high-quality South American reference genome named PvPAM (*P. vivax* Peruvian AMazon). The PvPAM chromosomes have a high level of completeness (telomeric repeats in 17/28 chromosomal ends), although 12 unassigned contigs remain. A variant analysis of publicly available WGS data shows that the PvPAM core genome represents South American and African isolates better than the PvP01 and PvW1 reference genomes. In the subtelomeric regions, the use of PvPAM as a reference genome resulted in a significantly lower number of variants for WGS data from all continents, outperforming PvP01 and PvW1.

## Results

### Construction of a high-quality reference genome from a low-input *P. vivax* patient DNA sample

A new reference genome was constructed from a monoclonal *P. vivax* patient sample originating from the Peruvian Amazon basin (Iquitos), which we named PvPAM (*P. vivax* Peruvian AMazon). Although the input sample contained a very low amount of parasite DNA (9 ng), the PacBio Ultra-Low Input protocol for library preparation allowed to sequence the *P. vivax* genome in high depth and enabled to build a high-quality reference genome for the South American Amazon region.

497 billion raw continuous long read (CLR) bases were sequenced, and after pre-processing, 24.5 billion high fidelity circular consensus sequencing (CCS) read bases were left, with reads being 10,670 bases long on average. When mapped to the PvP01 reference genome, this resulted in an average sequencing depth of 821x. This is well above the recommended 15x depth for assembly of a haploid genome [23], but due to the repetitive and AT-rich subtelomeric regions, higher depths are necessary to fully assemble the eukaryotic *P. vivax* genome.

Genome assembly and subsequent scaffolding resulted in 14 chromosomes, of which 4 were completely assembled, while the other 10 were scaffolds consisting of 2–4 contigs. The gaps between those contigs were patched based on the PvP01 and PvW1 reference genomes [9, 24]. The patches had a median length of 388 bases (range 41-2592 bases) (Supplementary Fig. 1), which indicates that the initial PvPAM assembly was of good quality and only few bases were uncovered. Further polishing of the genome with Illumina reads, obtained in parallel from the PvPAM isolate, introduced some PvPAM-specific changes in the patched regions. In addition to the 14 chromosomes, 12 contigs could not be uniquely assigned to any chromosome, although they were identified as subtelomeric based on their low GC content (< 25%) and presence of *vir* (variant interspersed repeat) genes.

**Fig. 1:**
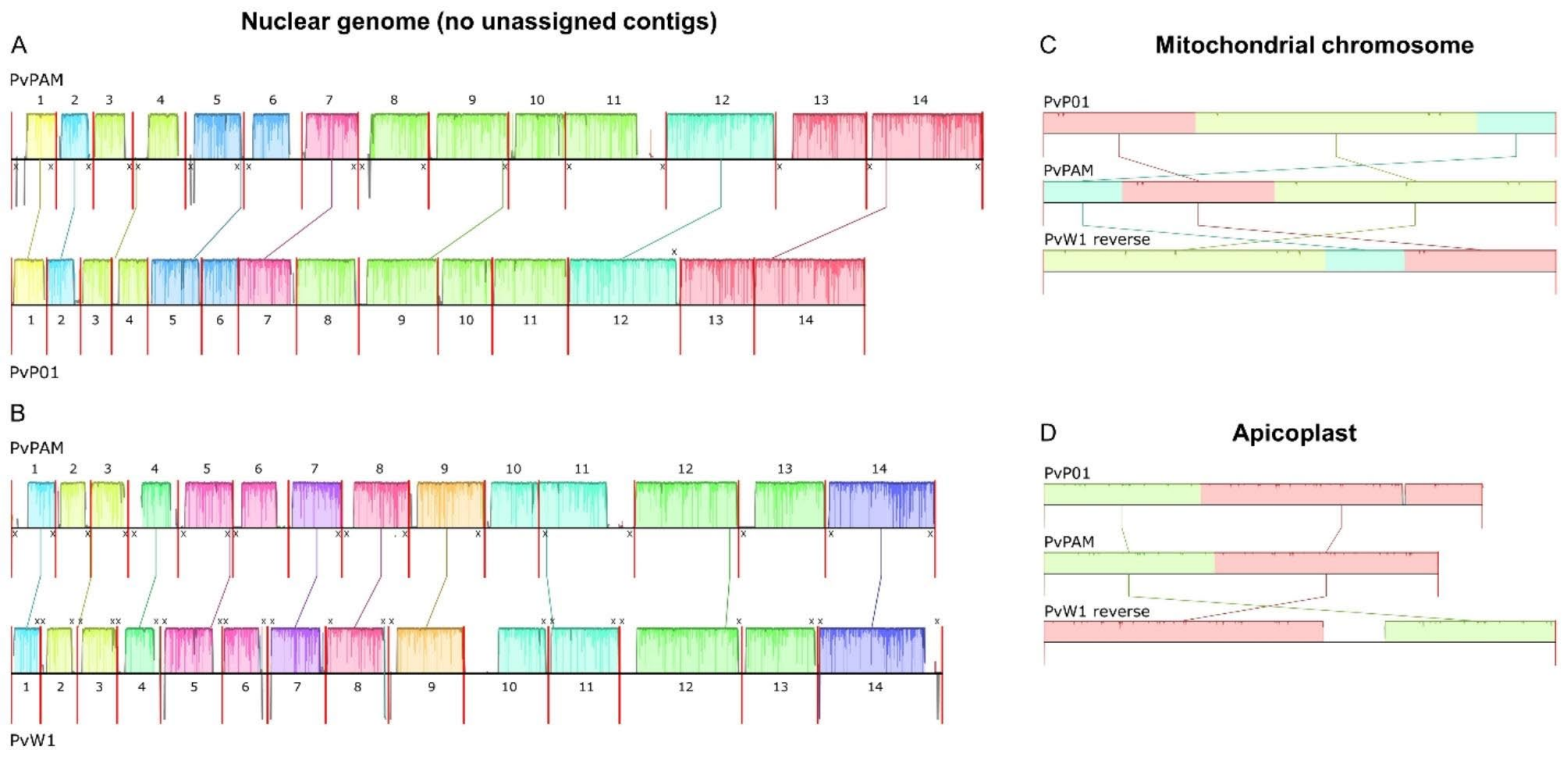
Whole genome alignments of PvPAM and PvP01 (**A**) and PvPAM and PvW1 (**B**), and chromosomal alignments of the mitochondrial (**C**) and apicoplast (**D**) genome. Coloured blocks indicate homologous regions, and their height indicates the level of similarity between both reference genomes. Blank regions indicate sequences that do not align between the two reference genomes. Chromosomes are separated by a red vertical line and are numbered. If a telomeric repeat is present at the chromosomal end, this is indicated by a cross. Unassigned contigs are not shown. The mitochondrial and apicoplast genomes (**C, D**) align at different locations for the three different reference genomes, which is due to their circular nature. The mitochondrial and apicoplast chromosomes of PvW1 were reverse complemented to match the orientation of PvP01 and PvPAM. Alignments were made with the Mauve Contig Mover (MCM) algorithm (**A, B**) and the Progressive Mauve algorithm (**C, D**) [53], using the PlasmoDB PvP01 v51 and PvW1 v60 genome

PvPAM chromosome-completeness was assessed by the presence of telomeric CCCT(A/G)AA repeats [25, 26], which indicate that the chromosome was fully assembled up to the 5’ or 3’ end. Telomeric repeats were only observed at one chromosomal end in PvP01, while they are present in 17/28 chromosomal ends in the PvPAM, indicating a higher number of completely assembled chromosomes (Fig. 1). Eight additional telomeric repeat sequences could be found in the 12 unassigned and subtelomeric PvPAM contigs.

### Structural comparison of PvPAM to the PvP01 and PvW1 reference genomes

PvPAM was compared on a structural and annotation level to the two highest quality *P. vivax* reference genomes existing to date: PvP01 [24] and the PacBio-based PvW1 [9]. These three reference genomes have overall similar genome sizes (Table 1). However, whole genome alignments of the reference genomes reveal that most PvPAM chromosomes contain longer subtelomeric regions than their PvP01 counterparts (Fig. 1A). This is because large parts of the PvP01 subtelomeric regions are divided over many short contigs that are not assigned to any chromosome, while the PvPAM reference genome is less fragmented. The subtelomeric regions of the PvW1 chromosomes are overall of similar length as in PvPAM (Table 1), but at the chromosome level, the subtelomeres often have different lengths or do not fully align (Fig. 1B). We determined core genome boundaries based on GC-content and sequence identity between PvPAM, PvP01 and PvW1. These borders show that the PvW1 and PvPAM chromosomes possess longer subtelomeric regions than the PvP01 chromosomes, due to higher contiguity in PacBio-based genomes (Table 1).

The PvPAM genome contains 6497 functional genes (i.e., genes not annotated as a pseudogene), a higher number than found in PvW1, but still less than in PvP01 (Table 1). Out of the 6497 functional genes, 1109 are not present in PvP01. Of those, 487 genes cannot be linked to any PvP01 gene through orthology, and are therefore new in the PvPAM reference genome (Supplementary Table 1). The number of PvPAM pseudogenes is higher than what was observed for PvP01 (4.7% vs. 2.7% of all genes). Pseudogenes are mainly located in the subtelomeres (89% of PvPAM and 95% of PvP01 pseudogenes), due to the dynamic nature of this region [14]. In the PvPAM mitochondrial and apicoplast genome, less functional genes were annotated in comparison to PvP01. In the apicoplast, this is partly due to its shorter length in PvPAM (Fig. 1D). However, the PvPAM apicoplast and mitochondrial genome are highly similar to PvP01 (Figs. 1C-D and 89.1% and 99.9% pairwise identity with MUSCLE, respectively), and missing gene annotations are attributed to a limitation of the Companion annotation tool.

**Table 1:** Comparison of the PvPAM, PvP01 (PlasmoDB, v51) and PvW1 (PlasmoDB, v60) assemblies. The assembly size includes unassigned contigs. The subtelomeric region size of chromosomes is based on the subtelomeric boundaries defined in the Methods section. Functional genes are all genes that are not annotated as pseudogenes. PvW1 pseudogenes are not annotated and therefore unknown. Mb = megabases, kb = kilobases

		PvPAM		PvP01		PvW1	
**Nuclear genome**							
Assembly size (Mb)		29.40		29.00		28.96	
GC content (%)		39.59		39.81		39.89	
Number of scaffolds assigned to a chromosome		14		14		14	
Number of unassigned contigs		12		226		3	
Size of unassigned contigs (Mb)		1.88		4.79		1.18	
Subtelomeric genome size (Mb)	Unassigned contig	9.41	1.88	9.01	4.76	8.98	1.18
	Part of chromosome		7.53		4.25		7.80
Number of functional genes		6456		6586		6119	
Number of pseudogenes		321		185		*NA*	
**Mitochondrial genome**							
Assembly size (bases)		5991		5989		5994	
GC content (%)		30.50		30.52		30.51	
Number of functional genes		2		35		2	
**Apicoplast genome**							
Assembly size (kb)		26.60		29.58		34.53	
GC content (%)		12.82		13.30		14.37	
Number of functional genes		39		55		54	
Number of pseudogenes		1		0		*NA*	

### PvPAM multigene families in subtelomeric or internally variable regions

Multigene families located at the subtelomeres or internally variable regions often play an important role in host-parasite interactions, immune response and pathogenicity, and can vary in (pseudo)gene number across isolates due to the dynamic nature of subtelomeres [4]. In particular, the *P. vivax* variant genes (*vir*) are known to be involved in immune invasion, and the *P. vivax* tryptophan-rich antigens (PvTRAg), *P. vivax* reticulocyte binding proteins (PvRBP) and *P. vivax* merozoite surface proteins (MSP) take part in invasion or red blood cell (RBC) binding.

In the PvPAM genome, 975 + 99 *vir* genes (functional genes + pseudogenes) were found, which is less than the 1093 + 116 in PvP01 and 1145 functional *vir* genes in PvW1. Remarkably, out of these 975 functional *vir* genes identified in PvPAM, only 370 have been transferred from the PvP01 reference genome through sequence similarity. The remaining 605 *vir* genes are newly predicted in PvPAM.

All 40 *PvTRAg* genes present in PvP01 were also found in PvPAM. No additional PvPAM *PvTRAg*’s were discovered based on othology groups (OrthoMCL) or the presence of the *Plasmodium* tryptophan-rich protein Pfam domain (PF12319). *PvTRAg36* (PVP01_0000140), which was reported to bind to the band 3 and basigin RBC receptors [27, 28], is annotated as a pseudogene in PvPAM. However, pseudogene annotation is based on prediction models. The utilization of an alternative translation start site and the splicing out of an intron (as seen in PvP01 *PvTRAg36*) would result in a fully functional PvTRAg36 protein. The more complete PvPAM assembly also enabled to locate 10 *PvTRAg*’s to chromosome 13, that were formerly annotated to the unassigned contigs ‘Transfer.PvP01_00_1.final’ (PvP01) and ‘CAJZCX010000017’ (PvW1).

Furthermore, the PvPAM genome contains all 5 full length *PvRBP* genes that are present in PvP01. Out of the 3 partial *PvRBP* genes that were identified in PvP01 and PvSalI, *PvRBP1-p1* (PVP01_0010770) and *PvRBP2-p2* (PVX_101590) are absent in PvPAM (Supplementary Table 2). *PvRBP2-p2* was reported to be present only in a subset of *P. vivax* samples, and is also absent in PvP01 [29]. Of the 3 *PvRBP* pseudogenes in PvP01, *PvRBP3* and the isolate-specific *PvRBP2e* [8] are identified in PvPAM. The PvP01 *PvRBP2d* pseudogene is replaced by 3 partial genes in PvPAM, while in PvW1 it consists of 2 partial genes (alignment in Supplementary Fig. 2, Supplementary Table 2). Further studies are required to confirm whether some isolates indeed show several shorter (partial) PvRBP2d proteins, or whether this is an annotation model artefact and in reality is one long pseudogene. No additional *PvRBP* genes were discovered in PvPAM based on orthology clusters or Pfam domains (PF16830, PF18515).

Additionally, multigene families that are located in tandem on hypervariable regions in the core genome, such as PvMSP3, PvMSP7 and the *P. vivax* serine-repeat antigens (PvSERA), have the potential to vary in number of genes [30]. The *PvMSP3* genes are highly polymorphic [31], and have been shown to have a varying number of genes at the center of the gene cluster [9]. Indeed, PvPAM contains two more centrally located *MSP3* genes (*MSP3.6*, *MSP3.7*) that are absent in PvP01 and PvW1, a feature it shares with the Central-American PvSalI. On the other hand, PvSalI’s *MSP3.4* is missing in PvPAM (Supplementary Fig. 3). There was no variation in the number of genes observed in the *PvMSP7* and *PvSERA* families between PvPAM and PvP01.

Although not located in the subtelomeres or internally variable regions, the erythrocyte binding–like (EBL) superfamily is also involved in invasion. Its best known member, the *P. vivax* Duffy binding protein (PvDBP), binds to the Duffy receptor to enable reticulocyte invasion [32]. *PvDBP* copy number variations have been associated with immune evasion [33, 34], but no additional *PvDBP* orthologs were identified in the PvPAM assembly. When PvPAM PacBio reads were mapped against the PvPAM genome, no increase in *PvDBP* coverage (indicative of a duplication) was observed either (Supplementary Fig. 4).

### PvPAM drug resistance-associated genes

Although drug resistance-associated mutations remain largely elusive in *P. vivax*, antifolate-resistance SNPs have been described for *dhps* and *dhfr* [35]. Antifolates are no longer recommended as a treatment in Peru, but the DHPS A383G and DHFR S58R and S117N resistance mutations are still highly prevalent in recently collected Peruvian samples [36–38]. As expected, these mutations are also present in the PvPAM genome. The PvP01 reference contains the same antifolate-resistance mutations as PvPAM, while PvW1 possesses a different combination of antifolate resistance SNPs (Supplementary Table 3). Although the PvSalI genome is geographically the closest to PvPAM, PvSalI is still fully sensitive to antifolate drugs (Supplementary Table 3), as it was collected almost 20 years before the first resistant cases were described [6, 39]. Overall, the PvPAM *dhps* and *dhfr* genes align well to their PvSalI, PvP01 and PvW1 homologs (≥ 98.3% and ≥ 99.8% sequence identity, respectively), except for an in-frame increase of tandem repeats upstream of the final *dhps* intron in PvPAM (Supplementary Fig. 5A,B). Alignments of the chloroquine resistance-associated genes *mdr1* and *crt* [35] of PvPAM, PvSalI, PvP01 and PvW1 show close similarities. PvPAM *mdr1* has 99.9% sequence identity to the *mdr1* genes in the 3 other reference genomes (Supplementary Fig. 5C). PvPAM *crt* showed a lower sequence identity of 96.8-99.2% to the *crt* sequence in the other reference genomes, due to PvPAM indels in introns 5, 9 and 12 that are caused by variations in tandem repeats (Supplementary Fig. 5D).

Gene duplications have been associated with antimalarial resistance as well, and the PvPAM PacBio reads were mapped to the PvPAM reference genome to check for increased coverage indicative of a potential duplication. In addition, a search for new orthology group members of resistance-associated genes was done. In South America, duplications of *crt* (potential chloroquine resistance), *dhfr* and *dhfs* (potential antifolate resistance), and *mrp1* (potential atovaquone resistance) have been described [36, 40], but were not observed in PvPAM (Supplementary Fig. 4). Mefloquine resistance-associated *mdr1* gene duplications have mainly been observed in Southeast Asia [24, 41–44], and were not found in the Peruvian PvPAM genome (Supplementary Fig. 4).

### South American samples mapped to PvPAM show more mapped reads and less read truncation

To investigate the difference in number of mapping reads between PvPAM, PvP01 and PvW1 as reference genome for South American WGS samples, WGS Illumina reads from 354 publicly available South American isolates and 63 non-South American isolates coming from Africa (N = 18) and Asia (N = 45), were mapped to the PvPAM, PvP01 and PvW1 reference genomes (overview of accession numbers and country of origin in Supplementary File 1).

**Fig. 2:**
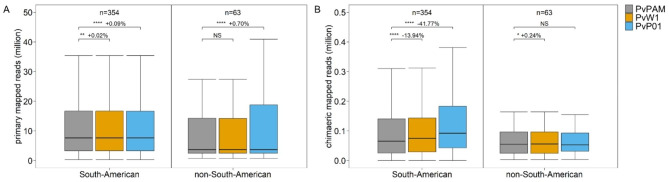
Mapping of South American reads to PvPAM results in more primary and less chimaeric reads. Box plots show the number of primary (**A**) and chimaeric (truncated; **B**) reads (in millions) mapped to the PvPAM, PvW1 or PvP01 reference genomes. Reads originate from South American and non-South American Illumina WGS samples, which are shown separately. Outliers (< quartile 1–1.5*interquartile range; > quartile 3 + 1.5*interquartile range) are excluded from the plot for visibility. NS non-significant, * p < 0.05, ** p < 0.01, *** p < 0.001, **** p < 0.0001 (paired Wilcoxon signed-rank test). Next to the significance value, the % increase (+) or decrease (-) of mapped reads to PvPAM relative to PvP01 or PvW1 is shown. It is not possible to compare number of mapped reads between the South American and non-South American region, since those reads originate from different samples. n = number of Illumina WGS samples

When South American WGS reads are mapped to each of the three reference genomes, differences in the number of mapped reads are small. 0.02-0.09% more reads align to PvPAM than to PvW1 or PvP01 (Fig. 2A; Wilcox *p* < 0.01 and < 0.0001 respectively). Additionally, 0.70% more non-South American reads are mapped to PvPAM than to PvP01 (Wilcox *p* < 0.0001), but there is no difference in the number of mapped reads between PvPAM and PvW1 (Wilcox *p* non-significant; NS) (Fig. 2A). The reference genome’s effect on read alignment becomes more apparent when chimaeric (truncated) reads are analysed separately. Mapping of South American reads to PvPAM results in 13.94-41.77% less chimaeric reads compared to mapping to PvW1 or PvP01 (Fig. 2B; Wilcox *p* < 0.0001 for both). This indicates a higher structural similarity between South American isolates and PvPAM than with other reference genomes. Indeed, when reads from non-South American samples are mapped to the 3 reference genomes, the number of chimaeric reads is comparable between PvPAM and PvP01 (Wilcox *p* NS), and 0.24% lower in PvW1 (Wilcox *p* < 0.05). This is in line with expectations, since 45 out of the 63 non-South American isolates are of Asian origin, and PvW1 originates from a Thai isolate that was sequenced with PacBio (Fig. 2B).

### PvPAM better reflects the South American variants

Since the PvPAM reference is derived from a Peruvian isolate, it likely resembles other South American isolates. Therefore, it is hypothesised that South American reads will contain fewer variants (SNPs, indels) when they are mapped to the PvPAM genome in comparison to PvP01 (Papua Indonesia) and PvW1 (Thailand). To investigate this, 354 publicly available Illumina WGS reads from South American samples (Peru, Brazil and Colombia; no other South American countries with > 10 WGS isolates were found in our public data search) were mapped to the PvPAM, PvP01 and PvW1 reference genomes and variant called. The same was done for 63 Illumina WGS samples from Africa (Eritrea, Ethiopia, Sudan, Uganda), South Asia (Afghanistan, India, Pakistan) and Southeast Asia (Cambodia, Malaysia, Myanmar, Papua New Guinea, Thailand, Vietnam). Since PvPAM has a slightly longer subtelomeric genome than PvP01 and PvW1 (9.4 Mb vs. 9.0 Mb) and since subtelomeric and core regions of the genome typically exhibit a different pattern of variant density (Supplementary Fig. 6), variants of the core and subtelomeric genome were analysed separately.

When WGS reads from South American samples are mapped to the PvPAM genome, the number of variants in the core genome is significantly lower in comparison to PvP01 or PvW1 (Table 2, Supplementary Fig. 7A,C). Interestingly, alignment of non-South American reads to the PvPAM genome still results in less core genome variants than when PvP01 is used as reference, but more core genome variants than with PvW1 as reference (Table 2, Supplementary Fig. 7A,C). When the non-South American samples are divided into specific geographical regions, it becomes clear that reads from African and South Asian samples still contain the least core genome SNPs when mapped to PvPAM. Reads from Southeast Asian samples on the other hand, contain less core genome SNPs when mapped to PvP01 or PvW1 in comparison to PvPAM (Table 2, Supplementary Fig. 7B). When indels are assessed, African reads still contain the least indels when mapped to PvPAM, but both South Asian as Southeast Asian reads now have the least indels when aligned to PvW1. In each case though, the use of PvP01 results in the highest number of indels, highlighting the advantage of structurally more correct PacBio references (PvW1, PvPAM) in the context of indels (Table 2, Supplementary Fig. 7D). Overall, PvPAM is the most suited reference genome for use with South American and African WGS reads to investigate core genome variants, while PvW1 or PvP01 are a better choice for Southeast Asian reads. Both PvW1 and PvPAM are suited for South Asian reads, depending on whether core genome indels or SNPs are the main interest.

In the subtelomeric genome, reads from South American samples again show lower numbers of variants when mapped to PvPAM in comparison to the other 2 genomes (Table 2, Supplementary Fig. 8A-D). Reads from non-South American samples, however, now also show less subtelomeric variants when mapped to PvPAM than when mapped to PvP01 or PvW1 (Table 2, Supplementary Fig. 8A,C), and this is true for all the different geographical regions assessed (Table 2, Supplementary Fig. 8B,D). This shows that the PvPAM subtelomeres are more similar to the subtelomeric regions of isolates from all continents than the PvP01 or PvW1 subtelomeres, although the PvPAM subtelomeres still resemble the South American subtelomeres the most (largest decrease in median number of variants – Table 2).

It can be concluded that the PvPAM reference reflects South American variants the best, and therefore, the generated vcf file containing variants of South American reads mapped to the PvPAM reference genome, can be of use for future research (available on the European Variation Archive (EVA)).

**Table 2:** Median number of variants depends on the used reference genome and region of origin. Numbers indicate the decrease (-) or increase (+) in median number of variants per 10,000 bp when Illumina WGS reads are mapped to PvPAM in comparison to PvP01 or PvW1. For example, South American reads show 5.26 core genome SNPs less per 10,000 bp when mapped to the PvPAM genome in comparison to the PvP01 genome. Values are shown separately for the core genome and subtelomeric genome, and variants are divided into SNPs and indels (insertions and deletions). * p < 0.05, ** p < 0.01, *** p < 0.001, **** p < 0.0001 (paired Wilcoxon signed-rank test). African samples originate from Eritrea (5), Ethiopia (5), Sudan (5) and Uganda (3), South Asian samples from Afghanistan (5), India (5) and Pakistan (5), and Southeast Asian samples from Cambodia (5), Malaysia (5), Myanmar (5), Papua New Guinea (5), Thailand (5) and Vietnam (5). N = number of Illumina WGS samples

	PvPAM vs. PvP01				PvPAM vs. PvW1				N
	SNPs		indels		SNPs		indels		
CORE GENOME									
**South America**	**+ 5.26**	****	**+ 2.41**	****	**+ 5.34**	****	**+ 2.10**	****	**365**
Peru	+ 5.61	****	+ 2.47	****	+ 5.63	****	+ 2.17	****	137
Brazil	+ 4.82	****	+ 2.40	****	+ 4.90	****	+ 2.10	****	152
Colombia	+ 4.40	****	+ 2.31	****	+ 4.56	****	+ 2.00	****	65
**Non-South America**	**+ 0.98**	*	**+ 0.94**	****	**-0.63**	*	**-0.03**	****	**63**
Africa	+ 2.15	****	+ 0.96	****	+ 0.80	****	+ 0.11	**	18
South Asia	+ 2.24	****	+ 1.03	****	+ 0.57	****	-0.04	*	15
Southeast Asia	-0.39	****	+ 0.25	****	-2.08	****	-0.66	****	30
SUBTELOMERIC GENOME									
**South America**	**+ 4.55**	****	**+ 0.45**	****	**+ 5.21**	****	**+ 0.48**	****	**365**
Peru	+ 1.63	****	+ 0.15	****	+ 1.67	****	+ 0.12	****	137
Brazil	+ 4.18	****	+ 0.30	****	+ 5.09	****	+ 0.43	****	152
Colombia	+ 5.18	****	+ 0.67	****	+ 7.41	****	+ 0.85	****	65
**Non-South America**	**+ 2.63**	****	**+ 0.18**	****	**+ 1.41**	****	**+ 0.05**	****	**63**
Africa	+ 1.61	****	+ 0.23	****	+ 0.82	****	+ 0.14	***	18
South Asia	+ 1.63	****	+ 0.18	****	+ 0.42	***	+ 0.06	*	15
Southeast Asia	+ 3.96	****	+ 0.34	****	+ 3.01	****	+ 0.16	****	30

## Discussion

This study presents the first high-quality South American *P. vivax* reference genome, named PvPAM. Although South American *P. vivax* isolates are genetically distinct from the other continents [12] and are the predominant cause of malaria in South America [1], no qualitative South American *P. vivax* reference genome existed until now. When compared to PvW1 and PvP01, PvPAM is the best suited reference genome for alignment of South American WGS reads. PvPAM was derived from a monoclonal *P. vivax* malaria patient from the Peruvian Amazon, and the isolate did not undergo culture adaptation prior to sequencing. Only 9 ng of DNA extracted from 100 µl of leukocyte depleted RBCs (1% parasitaemia) was used for PacBio sequencing, in contrast to the 107 ng obtained from controlled human infections for the construction of the PvW1 PacBio reference genome [9].

To assess the benefit of using a South American reference for read alignment of South American samples, PvPAM was compared to PvW1 and PvP01, the two other high-quality reference genomes existing for *P. vivax*. A small but significantly higher number of South American reads mapped to PvPAM in comparison to these other two references. More importantly, 14% and 42% less truncated reads were found compared to PvW1 and PvP01, respectively. For non-South-American samples, alignment to PvW1 and PvPAM resulted in a similarly high number of mapped reads, but use of the PacBio-based Thai PvW1 reference resulted in less truncated reads. Since read truncation is typically due to large structural variants (e.g. indels > 50 bp, translocations, inversions, copy number variations), this points towards continent-specific structural differences between the used reference genomes. In terms of number of mapped and truncated reads, PvPAM is well suited as a reference genome for South American WGS data, and is competitive with PvP01 and PvW1 for general use with WGS data from other continents.

The advantage of using a reference genome that is closely related to the genome of the sequenced isolate was further demonstrated by investigating the number of core genome variants. Alignment of South American WGS reads to the PvPAM reference showed the lowest number of core genome variants, indicating that South American samples resemble PvPAM most closely. Similarly, PvW1, originating from a Thai isolate, is the best reference to minimize the number of core genome variants in Southeast Asian samples. Alignment of African WGS reads to PvPAM still resulted in the lowest number of core genome variants, while alignment of South Asian reads to PvPAM and PvW1 produced the lowest number of core genome SNPs and indels, respectively. These findings mirror phylogenetic trees in which South American isolates are genetically most distinct from Southeast Asian isolates, but more similar to African and, to a lesser extent, South Asian isolates [12].

Alignment of WGS reads to PvPAM always resulted in the lowest number of subtelomeric variants, regardless of the sample’s country of origin. Interestingly, even Southeast Asian samples showed less subtelomeric variants when mapped to PvPAM compared to the Thai and Pacbio-based PvW1. However, *Plasmodium* subtelomeric regions remain largely elusive, and the extent to which the subtelomeric sequences vary between isolates and show geographical clustering is still uncertain. The subtelomeric regions of *P. falciparum* have been shown to vary in length across isolates [45], and similarly, PvPAM has longer subtelomeric regions than PvP01 or PvW1. The longer subtelomeres of PvPAM contain unique regions that are not present in either PvP01 or PvW1, which might result in a subtelomeric backbone that is better suited to align samples from all continents against. This could improve accuracy of read mapping and thus lead to lower variant densities.

The high-quality and longer subtelomeric regions of the PvPAM genome provide new insights into the subtelomeric gene families and their diversity. A comparison of *vir* genes between PvP01 and PvPAM reveals that both have a high number of unique functional *vir* genes that are absent in the other genome (723 and 605, respectively). It is possible that this high amount of uniquely identified *vir* genes are of the same origin as the *vir* genes found in PvP01, but that differences introduced by subtelomeric recombination caused the Companion annotation tool to identify many *vir* genes as novel. This could be due to geographical differences between PvP01 and PvPAM, but it is also possible that there is an inherent variability in *vir* genes, even across isolates from the same region. Other subtelomeric gene families did not show a gain or loss of gene members in PvPAM and appear to be more stable than the *vir* family. However, more *P. vivax* assemblies with high-quality subtelomeres are needed to study variation of *vir* genes and other subtelomeric multigene families in depth. Some subtelomeric genes, such as the *PvTRAg25*-*PvTRAg33* cluster, could for the first time be located to a chromosome. On the other hand, 12 subtelomeric PvPAM contigs could not be assigned to any chromosome based on PvP01 or PvW1 sequence similarity.

In this study, the core genome of PvPAM as well as PvP01 and PvW1 was defined based on GC-content and pairwise alignment between these three genomes. Although no standard definition is available for subtelomeric-core genome boundaries, GC-content has been used earlier as a marker for *P. vivax* subtelomeres [4]. The provided core genome regions could be further refined by comparing positionally conserved genes across different *P. vivax* isolates, as was done for *P. falciparum* isolates [45]. This, again, would require multiple high-quality *P. vivax* subtelomere assemblies that are currently not available. However, the here used combination of long-read sequencing and a low-input material approach, might facilitate assembly of *P. vivax* field isolates from blood volumes as small as a finger prick. In addition, Oxford Nanopore’s portable sequencer could become a quick and easy to use alternative for PacBio sequencing [46], given further improvements in accuracy and DNA input requirements.

Finally, genome annotation was performed using the Companion annotation tool [47], which provides an initial automatic annotation by transferring PvP01 annotations and conducting ab initio gene identification. However, due to limitations in the current version of this tool, we observed some missing genes in the mitochondrial and apicoplast genome of PvPAM compared to PvP01. Additional manual curation and an improved future version of the Companion tool will further enhance the PvPAM annotation. The PvPAM genome and new annotation releases will be made available on PlasmoDB.

## Conclusions

In summary, the PvPAM reference genome is the best choice to align South American WGS data to, highlighting the importance of using a continent-specific reference. Furthermore, it performed well for African and, to a lesser extent, South Asian WGS data. When studying subtelomeric regions, PvPAM proved to be the most suited reference genome, regardless of the sample’s continent of origin. Overall, the PvPAM reference genome will improve the quality of genomic analyses conducted on South American samples or subtelomeric genomes.

## Methods

### Collection and processing of *P. vivax* patient blood samples

*P. vivax–*infected blood was collected in 2019 from adult patients (≥ 18 years) with acute *P. vivax* infection from the Peruvian city of Iquitos and neighbouring communities. All *P. vivax* cases were diagnosed by light microscopy. 10–20 mL blood samples were collected in lithium-heparin tubes from patients with single *P. vivax* infections with parasite densities > 0.1% and gametocyte proportion < 50%.

Blood samples from patients with *P. vivax* infection were processed within 6 h of collection. Leukocytes and platelets were depleted using cellulose columns [48]. Then, 100 µl of *P. vivax–*infected RBCs were stored at 50% haematocrit for future short- and long-read whole genome sequencing (WGS). The remaining blood was used for other purposes.

### DNA extraction and size selection of Pv01-19

Out of the 22 collected samples, sample Pv01-19 contained the highest parasitaemia of 1.0% (approximately 90% ring stages), and was therefore selected for long and short-read WGS. DNA was extracted from the stored 100 µl leukocyte-depleted RBCs using the QIAamp DNA mini kit (Qiagen), following the manufacturer’s instructions. After elution in 50 µL AE buffer, 0.46 ng/µl DNA was obtained. Fragments < 3 kb were removed with Ampure PB beads (2.2 V of 40% beads), and DNA was eluted in 50 µl PacBio elution buffer, resulting in 0.21 ng/µl DNA. Input fragment target size for the PacBio ultralow input protocol is 8–10 kilobases (kb). Since the concentration of Pv01-19 was too low (< 1 ng/µl), no Fragment Analyzer (Agilent) profile could be made. However, Fragment Analyzer profiles of higher concentration samples that were similarly stored fell within the 8–10 kb range, which is why no further shearing was applied on the Pv01-19 DNA.

### PacBio sequencing of Pv01-19

9 ng of DNA was used for library preparation with the SMRTbell Express Template Prep Kit 2.0, following the ultralow input protocol (version 01, August 2020). This protocol includes a 13-cycle PCR step to amplify the low amount of input DNA, using reagents from the SMRTbell gDNA Sample Amplification Kit (master mixes A and B cover different GC compositions). For the mix A PCR, 3 additional cycles were done to increase the yield. The final library was size-selected for fragments > 6,800 bases with a BluePippin device (Sage Science), since the Fragment Analyzer profile showed a peak at 8,304 bases. After clean-up, a 14.3 ng/µl library was obtained, with a fragment length peak at 8,341 bases (Femto Pulse, Agilent). The Femto Pulse trace showed no signs of dimer or overamplification.

SMRTbell templates were annealed with Sequencing Primer v4 and bound to a polymerase using the Sequel II binding Kit 2.0. SMRT Link settings for sequencing were entered according to the ultralow input protocol (CCS mode, Iso-Seq Experiment enabled, Iso-Seq Version Express to enable ProNex Cleanup workflow, with ProNex cleanup of 50% anticipated). Next, the library was loaded onto an 8 M SMRT cell and sequenced on a Sequel II with 2 h pe-extension time and 30 h movie time (Earlham Institute, Norwich). 6.1 million continuous long reads (CLR) were generated, resulting in a total number of 497 billion sequenced bases.

### Illumina sequencing of Pv01-19

To be able to polish PacBio sequencing errors, 1 ng DNA of the Pv01-19 sample was sent for Illumina sequencing (GenoScreen, Lille). The library was prepared with the Nextera XT DNA Sample Prep Kit (Illumina) following the manufacturer’s protocol, and sequenced on an Illumina NovaSeq 6000 platform with 2 × 150 bp paired-end reads. 68.3 million raw reads were generated, resulting in 10.3 billion sequenced bases.

### Pre-processing of PacBio reads

Reads were pre-processed with the PacBio secondary analysis tools available on PacBio Bioconda (https://github.com/PacificBiosciences/pbbioconda). Raw CLR subreads were converted to HiFi circular consensus sequencing (CCS) reads with pbccs v6.0.0 (--hifi-kinetics). During this process, 43% of the reads were lost, mainly due to misalignment of subreads from the same CLR read. To retain the ‘pw’ tag necessary for future Arrow assembly polishing, the ccs-kinetics-bystrandify tool (v2.0.0) from pbbam was used. PCR-adapters were trimmed with lima (--same --ccs --min-score 80), during which 5.6% of the CCS reads were lost. Next, duplicate removal with pbmarkdup v1.0.2 (--rmdup) caused another 5.6% of trimmed CCS read loss. This resulted in a final number of 24.5 billion CCS bases, with an average read length of 10,564 bases. To remove human reads, reads were mapped to the human genome (GRCh38) with the minimap2 wrapper pbmm2 v1.8.0 (--preset CCS --sort --unmapped), after which the unmapped reads were selected with samtools view (-f 4). Human reads represented only 0.18% of all CCS trimmed and non-duplicate bases.

Presence of contaminating species was checked with a Kraken2 run (--confidence 0.1; v2.0.9-beta) [49], during which the pre-processed and human-filtered reads were taxonomically classified with the PlusPF (09/08/22) database. No contaminants were identified that represented ≥ 0.01% of the reads.

Next, the pre-processed reads were mapped to the PvP01 reference genome (PlasmoDB, v51) with pbmm2 v1.8.0 to estimate the depth and check the similarity with the PvP01 reference genome.

### Assembly of the PvPAM reference genome

The pre-processed PacBio reads were *de novo* assembled into a draft genome with Canu v2.0 (-pacbio hifi) [50]. Next, contigs were scaffolded onto the PvP01 (PlasmoDB, v51) and PvW1 (PlasmoDB, v60) chromosomes using RagTag v2.1.0 (scaffold command, no prior correction done to prevent loss of strain-specific information) [51], and additional verification with minimap2 (-ax map-pb). When a contig was scaffolded differently on PvP01 as compared to PvW1, it was manually checked to which chromosomal location the contig fit best or if it had to be removed from the scaffold due to low confidence. 12 contigs could not be scaffolded confidently to any chromosome and remained unassigned. No contigs were scaffolded to the mitochondrial chromosome, due to its small size of approx. 6 kb (smaller than mean read length). Therefore, CCS reads that aligned (pbmm2) to the PvP01 or PvW1 mitochondrial chromosome were selected, duplicate removed (pbmarkdup), and assembled separately with Canu. Duplicated regions (circular chromosome) were manually removed.

Gaps between contigs of the same scaffold were then manually patched based on the PvW1 and PvP01 reference genome, using Geneious Prime (2022 release). PvW1 was preferentially used for patching, except when the contig ends only aligned to PvP01 (only the case for one patch on chromosome 2). Dotplots were made to check for large duplications due to assembly errors or due the circular mitochondrial and apicoplast chromosomes, but none were observed.

The assembly was polished based on the pre-processed CCS reads with 3 Arrow iterations (GCpp v2.0.2, https://github.com/PacificBiosciences/gcpp), introducing 5,967 changes to the assembly. This was followed by three iterations of Pilon (v1.23) short read polishing, resulting in 970 more changes. For each round of Pilon polishing, Illumina reads were mapped to the assembly with bwa mem v0.7.17, sorted on coordinates (samtools sort), duplicate reads were removed with Picard’s MarkDuplicates v2.22.0 (REMOVE_DUPLICATES = true), and confidently mapped reads (mapping quality = 60, no secondary reads) were selected with samtools view (-q60 -F256). The latter is done to prevent the shorter Illumina reads from misaligning to repetitive regions and incorrect polishing.

Completeness of the assembly was checked with Busco v5.4.4 [52]. Number of single-copy, fragmented and missing BUSCO’s (Benchmarking Universal Single-Copy Orthologs) are within the same range for PvPAM, PvP01 and PvW1 (Supplementary Table 4).

Finally, the PvPAM assembly was annotated with Companion v1.0.2 [47]. Contiguation was turned off, reference proteins were aligned to the target sequence, and RATT was used for gene transfer (on species level) from the PvP01 reference genome. For other parameters, default settings were used.

### Defining the subtelomeric-core genome boundaries

Boundaries between the core genome and subtelomeric regions were here defined based on GC content. GC content was calculated per 1 kb window (sliding at steps of 100 bases) with bedtools v2.29.2 (makewindows, nuc). In R v4.2.1, draft subtelomeric boundaries were defined from both sides of the chromosome as the first window of a 10 kb region (100 consecutive windows) for which GC rose consistently above 35%. These draft core genome regions were then extracted from the PvPAM assembly with samtools faidx. Per chromosome, the PvPAM draft core region was aligned to the corresponding PvP01 (PlasmoDB, v46) and PvW1 chromosomes with Progressive Mauve (default settings) in Geneious Prime [53]. The part of the draft core region that aligned to the 2 other reference genomes was considered to be the final core genome region, and these regions are shown in Supplementary File 2.

Unassigned contigs were categorised as subtelomeric or core based on their GC content. If the GC content was below the mean GC content of the core and subtelomeric regions of the 14 chromosomes (as determined above), the contig was considered subtelomeric. This was the case for all contigs of PvPAM and PvW1, and for 207/226 contigs of PvP01 (Supplementary File 2).

### Mapping of publicly available WGS data to PvPAM, PvP01 and PvW1 and variant calling

To be able to compare mapping quality and variants between the PvPAM, PvP01 and PvW1 genomes, and to construct a South American vcf (variant call format) file, 354 South American (152 Brazil, 65 Colombia, 137 Peru) and 63 non-South American (5 Afghanistan, 5 Cambodia, 5 Eritrea, 5 Ethiopia, 5 India, 5 Malaysia, 5 Myanmar, 5 Pakistan, 5 Papua New Guinea, 5 Sudan, 5 Thailand, 3 Uganda, 5 Vietnam) Illumina WGS samples were obtained from public databases [7, 11, 12, 30, 36, 38, 54–61] and newly sequenced samples from Peru and Brazil (SRA BioProjects PRJEB59758 and PRJNA934307, respectively). Supplementary File 1 gives an overview of the used accession numbers, their country and the study they originate from.

Raw reads were mapped to the human reference genome (GRCh38) with bwa mem v0.7.17 and coordinate sorted with samtools, after which the non-human reads that did not map as a pair were selected (samtools view -F 2) and converted to the fastq format again (samtools fastq). Those were then mapped to the PvPAM, PvP01 (version 46) or PvW1 reference genome with bwa mem, coordinate sorted, and duplicate marked with Picard MarkDuplicates v2.22.0. Per sample, the median alignment score was extracted using an awk loop, and number of primary and chimaeric reads were extracted with samtools view -c (-F 256 -F 2048 -F 1024 and -f 2048 -F 1024 respectively). The same was done on only the core genome of those same mapped files, as defined in previous section (Supplementary File 2).

Next variants were called using the GATK (v4.1.4.1) HaplotypeCaller (-ERC GVCF), and resulting gvcf files samples were combined (GATK CombineGVCFs) and converted (GATK GenotypeGVCFs) to a South American and a non-South American vcf file. Each vcf was split into a SNP and indel vcf (GATK SelectVariants), after which filter fields were defined according to the GATK golden standard with VariantFiltration (for the SNP vcf: QD < 2.0, QUAL < 30.0, SOR > 3.0, FS > 60.0, MQ < 40.0, MQRankSum<-12.5, ReadPosRankSum<-8.0; for the indel vcf: QD < 2.0, QUAL < 30.0, FS > 200.0, ReadPosRankSum<-20.0). Only variants that passed the filters described and did not have > 50% of missing genotypes (bcftools view -i ‘F_MISSING < 0.5’) were retained in the vcf files. Variants were annotated with SnpEff v4.3t and the -stats summary was used to compare the variant rate per base, mean allele frequency and mean indel length between the three used reference genomes. This was repeated on the same vcf files for which first the core genome was extracted (core genome regions in Supplementary File 2). Vcf files are made available under Study PRJEB60575 on the European Nucleotide Archive, and Supplementary File 1 links the WGS samples names used in the vcf to their accession numbers.

### Multiplicity of infection (MOI) estimation of Pv01-19

To estimate the MOI of Pv01-19, its Illumina reads were mapped to PvP01 (PlasmoDB v46) with bwa mem v0.7.17 and coordinate sorted (samtools sort), and duplicate reads were flagged with Picard’s MarkDuplicates v2.22.0. The same was done for Illumina reads from 15 other Peruvian samples collected at the same location and in the same way (BioProject PRJNA853729) [57], to provide samples for comparison to the MOI estimation tools. Pv01-19 was variant called together with the 15 other Peruvian samples following the same pipeline as described in previous section for the public data samples. Core and non-hypervariable regions as defined by Pearson et al. (2016) were extracted from the SNP vcf with bcftools tabix, after which estMOI v1.03 [62] and the moimix R package [63] were ran. Both tools estimated a MOI = 1 for Pv01-19.

### Statistical analysis

Non-parametrical paired Wilcoxon signed-rank tests were performed in R v4.2.1 [64].

### Electronic supplementary material

Below is the link to the electronic supplementary material.

Supplementary Material 1: Supplementary figures 1-8, Supplementary Tables 1-4

Supplementary Material 2: Supplementary file 1

Supplementary Material 3: Supplementary file 2

Supplementary Material 4: Supplementary file 3

Supplementary Material 5: Supplementary file 4

## Data Availability

The annotated reference genome and raw PacBio and Illumina reads used for its construction are available under Study PRJEB59758 in de European Nucleotide Archive (https://www.ebi.ac.uk/ena/). Additionally, the reference genome (.fasta.gz) and annotation file (gff3 format) are made available in Supplementary Files 3 and 4, respectively. Newly generated WGS Illumina reads from Peruvian (N = 7) and Brazilian (N = 21) samples are made available under BioProjects PRJNA853729 and PRJNA934307 in the Sequence Read Archive, respectively (https://www.ncbi.nlm.nih.gov/sra). The variant files (.vcf) containing SNPs and indels of publicly available South American WGS Illumina data mapped to the PvPAM genome, are available under Study PRJEB60575 in the European Nucleotide Archive (https://www.ebi.ac.uk/ena/). Supplementary File 1 links the sample names used in the vcf to their accession numbers.

## Abbreviations

CCS: Circular consensus sequencing
CLR: Continuous long read
EBL: Erythrocyte binding–like superfamily
Indel: Insertion or deletion
Kb: Kilobases
Mb: Megabases
NS: Non-significant
ONT: Oxford Nanopore technology
PvDBP: *P. vivax* Duffy binding protein
MSP: *P. vivax* merozoite surface proteins
PvRBP: *P. vivax* reticulocyte binding proteins
PvSERA: *P. vivax* serine-repeat antigen
PvTRAg: *P. vivax* tryptophan-rich antigens
*Vir*: *P. vivax* variant genes
PacBio: Pacific Biosciences SMRT technology
PvPAM: *P. vivax* Peruvian AMazon
RBC: Red blood cell
Vcf: Variant call format
WGS: Whole genome sequencing
